# Understanding Consumption Reduction Through the TPB: Moderating Effects of the Need for Evaluation and Self-Referencing Individual Differences

**DOI:** 10.3390/jintelligence12110119

**Published:** 2024-11-18

**Authors:** Shiva Ghorban Nejad, Heidi Victoria Skeiseid, Lukasz Andrzej Derdowski

**Affiliations:** NHS—Department of Leadership and Service Innovation, University of Stavanger, 4021 Stavanger, Norway; heidi.v.skeiseid@uis.no (H.V.S.); lukasz.a.derdowski@uis.no (L.A.D.)

**Keywords:** consumption reduction, environmental sustainability, TPB, individual differences, need for evaluation, self-referencing, moderating effect

## Abstract

Limited research has focused on consumption reduction as one potential pathway to meet sustainable development goals. This paper investigates consumers’ intentions to undertake consumption reduction through the lens of an extended theory of planned behavior (TPB), where selected individual differences, namely the need for evaluation (NE) and self-referencing (SR), are given considerable attention. In total, 226 respondents participated in this web-based survey study. The results from structural equation modeling analysis confirm that the extended theory of planned behavior effectively explains consumers’ intentions to undertake consumption reduction. Notably, the individual differences of the NE and SR each uniquely moderate the relationships within the TPB model. This study provides a theoretical contribution by integrating the selected moderators (i.e., the NE and SR) into the TPB framework, increases the TPB’s predictive power, and further provides a novel understanding of the underlying influences of individual differences on consumers’ intentions to undertake consumption reduction for the benefit of the environment. Moreover, the findings offer practical implications for policymakers and social marketers in designing tailor-made interventions and consumption reduction strategies by considering the important role of individual differences.

## 1. Introduction

Global consumption patterns show no signs of significant change. The recent United Nations climate report warns of the increasing pressure on resources and material footprints in affluent countries, which is ten times the material footprints in low-income countries (UN DESA 2023; Global Footprint Network 2022). Researchers and policymakers have long favored technological developments, like, e.g., Artificial Intelligence (AI) or the blockchain (Singh et al. 2020), and various green initiatives to tackle globally acknowledged environmental issues caused by consumption (Tanner and Wölfing Kast 2003; White et al. 2019; Dauvergne 2022). However, mounting evidence suggests that these undertakings may happen to backfire and further supercharge consumerism as many consumers tend to keep their consumption levels high (Dauvergne 2022; Visser and Schoormans 2023) due to, for instance, a rebound effect (Binswanger 2001). In response to these observations, scholars suggest the concept of environmentally motivated consumption reduction (EMCR) as one additional pathway to meet sustainable environmental goals (Egea and de Frutos 2013; Nejad et al. 2024; Peattie and Peattie 2009). EMCR is defined as individuals’ voluntary reduction in consumption levels to benefit the environment (Egea and de Frutos 2013). Building on the consumption reduction perspective, this paper argues that in addition to existing knowledge on how to promote sustainable consumption choices and behaviors, there is a need for a greater understanding of the underlying factors influencing individuals’ intentions to generally consume less for the sake of the natural environment.

Among the theoretical frameworks applied for understanding a pro-environmental behavior, such as EMCR, the theory of planned behavior (TPB) is a well-established model (Yuriev et al. 2020). Essentially, it indicates that individuals’ attitudes toward behavior, subjective norms, and perceived behavioral control are key predictors of ones’ behavioral intentions and subsequent behaviors (Ajzen 1991). However, despite its widespread application, the TPB has shown certain limitations in predicting pro-environmental intentions, explaining only between 27% to 39% of the variance in respondents’ intentions (see meta-analysis by Armitage and Conner 2001; Bamberg and Möser 2007). Recognizing the gap, researchers suggest extending the TPB by incorporating additional variables into the model (Yuriev et al. 2020; Heidbreder et al. 2020; Ansu-Mensah and Bein 2019). Haugtvedt et al. (2018) highlight the importance of individual differences in understanding consumer behaviors and suggest scrutinizing individual differences to not only extend the current knowledge base of existing theoretical frameworks, but also to deepen our insights into what drives and modifies individuals’ attitudes and decision-making processes.

Prior studies have examined the role of various individual differences and personality traits, such as the Big Five, a need for cognition, and self-monitoring, in affecting consumers’ consumption reduction behavior (Armstrong Soule and Sekhon 2022; Ghorban Nejad and Hansen 2021; Hüttel et al. 2020). They have evidenced that individual differences, such as personality traits, matter when one attempts to explain and/or predict consumers’ pro-environmental behaviors (e.g., Hopwood et al. 2022) and arguably EMCR. Moreover, a recent review article on individual-level consumption reduction highlights the scarcity of research on the underlying influences of cognitive processes and individual differences such as personality traits in consumers’ engagement in consumption reduction practices (Nejad et al. 2024). This study builds on the aforementioned strand of research and places considerable attention on two unique individual differences. That is, the need for evaluation (NE) and the self-referencing (SR) (Haugtvedt et al. 2018). At heart, the NE reflects individuals’ tendency for evaluative responding when making judgments or decisions (Jarvis and Petty 1996) and the SR reflects the degree to which individuals differ in their tendency to draw on aspects of the self and their prior experiences (Burnkrant and Unnava 1995; Haugtvedt 1994). Different from broader personality dimensions like the Big Five, these two traits specifically highlight individuals’ evaluative responses and self-related decision processes, which are essential for understanding why consumers behave in a certain way (Hansen and Sand 2008; Hansen et al. 2011; Haugtvedt et al. 2018).

Following the prior research findings and gaps and to advance the existing knowledge base on the EMCR, this paper adopts and empirically tests hypotheses derived from an extended TPB, where selected individual differences (i.e., the NE and SR) are given considerable attention. Thus, the contribution of this article is twofold. First, it extends the existing body of research on the topic of environmentally motivated consumption reduction in the context of an affluent society (Norway). Secondly, this scholarly endeavor augments the TPB model by adding unique moderating variables, thereby increasing the explained variance and enhancing our understanding of complex interrelations underlying consumers’ EMCR intentions.

## 2. Theoretical Background

### 2.1. Environmentally Motivated Consumption Reduction (EMCR)

The concept of pro-environmental consumption behavior refers to purchasing behaviors that are less harmful to the environment and encompasses actions ranging from shifting to a green consumption to a more radical consumption reduction (Steg and Vlek 2009; Schultz and Kaiser 2012). The EMCR falls under the consumption reduction path of the pro-environmental behavior spectrum and is defined as individuals’ voluntary reduction in consumption levels for the benefit of the environment (Egea and de Frutos 2013). The main characteristic of EMCR is that consumers intentionally make more significant changes in their consumption behavior and lifestyles rather than simply following a low-effort pro-environmental behavior such as recycling or purchasing eco-friendly products (Lembregts and Cadario 2024). For instance, consumers who are driven by environmental concerns may intentionally reduce a number of their leisure travels by staying at home (e.g., in line with staycation tourism (Pichierri et al. 2023), voluntarily reduce their overall meat consumption, or restrain oneself from purchasing a new item (see Ghorban Nejad and Hansen 2021; Miguel et al. 2020; Joanes 2019; Joanes et al. 2020).

In EMCR research, the majority of studies have explored the antecedents of consumption reduction intentions, examining how attitudes, norms, and values influence consumers’ willingness to engage in such behaviors (Ansu-Mensah and Bein 2019; Borusiak et al. 2022; Egea and de Frutos 2013; Joanes 2019; Russo et al. 2023). A parallel stream of research has explored the role of sociodemographic factors, revealing, for instance, that younger and more educated individuals are more inclined toward EMCR practices (Boto-García and Bucciol 2020; Culliford and Bradbury 2020). Additionally, strategies to encourage consumption reduction, including nudging, gamification, and incentives, have been investigated, indicating the potential to influence consumer EMCR behavior that corresponds to greater environmental responsibility (Mundt et al. 2020; Mulcahy et al. 2021; Rajapaksa et al. 2019). In the following sub-section, we elaborate on how we adapted concepts central to the TPB model to consumers’ EMCR intentions and extended the presented model by incorporating two moderating factors: the NE and SR personality differences.

### 2.2. The Theory of Planned Behavior (TPB)

The TPB is a well-known socio-psychological model that explains and understands individuals’ behavioral intentions, environmental behaviors, and behavioral changes (Ajzen 1991). Earlier studies in pro-environmental research adopted the TPB model to understand individuals’ pro-environmental behavior (e.g., Onel 2017), and more specifically EMCR behavior (Ansu-Mensah and Bein 2019; Heidbreder et al. 2020). The fundamental rationale behind the model states that consumers’ attitudes toward behaviors (one’s positive and negative evaluations), subjective norms (the social pressure that one perceives from their significant others), and perceived behavioral control (the control one has over undertaking a behavior) determine consumers’ intention to undertake a particular behavior (Ajzen 1991). It further considers intention as the closest predictor of one’s behavior. In other words, if someone intends to undertake a behavior, then one is more likely to act on that specific behavior (Ajzen 1991). Therefore, when employing the TPB model, it is a common and widely acknowledged practice to measure intentions as a proxy predictor of behavior rather than the actual behavior itself that typically requires more sophisticated research designs (Yuriev et al. 2020). Below, we explain the TPB core variables and the hypotheses illustrating the main effects, and then we proceed by providing an overview of the available literature supporting the development of hypotheses addressing the moderating influence of the proposed personality traits.

#### 2.2.1. Attitude Toward the Behavior

Ajzen (1991) highlights that favorable or unfavorable attitudes toward specific behaviors significantly influence the formation of behavioral intentions and subsequent behavior. This suggests that when an individual holds a favorable attitude towards engaging in a specific behavior, it enhances one’s motivation and intention to undertake the behavior in question and vice versa (Ajzen 1991). Earlier studies found that pro-environmental behavior, and EMCR behavior in particular, can be undertaken by consumers’ positive attitudes toward pro-environmental behavior (Ansu-Mensah and Bein 2019; Borusiak et al. 2022; Gansser and Reich 2023). This implies that a positive attitude toward such behaviors leads indirectly to actual pro-environmental behavior through one’s intentions (Onel 2017).

For example, in the energy context, positive attitudes toward electricity conservation are positively related to individuals’ intention to conserve electricity (Ansu-Mensah and Bein 2019). In the food and diet context, the literature suggests that the attitude toward the impact of environmentally-driven meat consumption reduction positively relates to the intention to reduce meat consumption due to environmental concerns (Borusiak et al. 2022; Weibel et al. 2019). Generally, it has been evidenced on multiple occasions that attitudes toward behavior are important in predicting consumers’ behavioral decisions and intentions (Weibel et al. 2019). Thus, following the aforesaid logic that has been exemplified by a broader set of consumption reduction practices, it can be argued that consumers’ attitude toward pro-environmental behavior is positively related to their consumption reduction intentions. Formally, it goes as follows:

**Hypothesis 1.**  *There is a positive relationship between individuals’ attitudes toward pro-environmental behavior and their intentions to undertake EMCR behavior.*

#### 2.2.2. Subjective Norm

Another factor that has consistently been found to positively relate to consumers’ intentions to embrace pro-environmental behavior is a person’s self-evaluation of subjective norms. Ajzen (1991) defined a subjective norm as the “perceived social pressure to perform or not to perform the behavior” (p. 188). In the context of pro-environmental behavior, if one perceives that their significant others (e.g., family, friends, etc.) approve of or encourage the individual to engage in a certain behavior that benefits the environment, then one is more inclined to and intends to undertake that specific behavior. Subjective norms have been examined broadly in consumers’ pro-environmental behavior studies (Onel 2017), including EMCR behavior (Borusiak et al. 2021; Canova and Manganelli 2020). For instance, Canova and Manganelli (2020) found that one’s perception of the approval of the people who are important to them to engage in energy consumption reduction that benefits the environment is positively related to one’s intention to reduce energy consumption. In the light of available findings, it is evident that consumers’ behavioral intentions toward reduction are not only influenced by their attitudes, but also by their perception of what their significant others think or approve. Thus, in the context of this study, the following is hypothesized:

**Hypothesis 2.**  *There is a positive relationship between individuals’ subjective norms and their intentions to undertake EMCR behavior.*

#### 2.2.3. Perceived Behavioral Control (PBC)

Ajzen (1991) refers to perceived behavioral control (PBC) as the “perceived ease and difficulty of performing the behavior” (p. 188), and it is presumed that this perception reflects upon the degree of control over performing the behavior and expected challenges (Ajzen 1991). For example, a consumer who intends to reduce private car usage by shifting to public transportation for environmental reasons requires access to some specific services in the first place (e.g., proximity and availability of public transport stations, and convenience in using public transport also under extreme weather conditions). These perceived obstacles or expected challenges may cancel out individuals’ intention or motivation to undertake a pro-environmental behavior, just as Armitage and Conner (2001) noted that people tend to participate in behaviors they consider attainable. In other words, while attitude and subjective norms relate to the motivation behind an individual’s behavior, PBC accentuates the aspect of control over the behavior and the ability to act on those behaviors (Ajzen 1991). Therefore, PBC has an important role in predicting and explaining behaviors (Ajzen 1991) and it has frequently been found to represent a strong factor influencing consumer pro-environmental intentions (Canova and Manganelli 2020). Several prior studies have found a significant relationship between PBC and behavioral intentions; for example, the intention to purchase domestic foods (Vabø and Hansen 2016) or the intention to reduce energy consumption for environmental sustainability (Canova and Manganelli 2020). In contrast, Onel (2017) found no association between consumers’ PBC and their intentions to purchase pro-environmental products. The examination of the existing literature paired with the scrutiny of the reasoning behind the TPB theory prompted us to formulate the following hypothesis in the context of consumption reduction:

**Hypothesis 3.**  *There is a positive relationship between individuals’ PBC and their intentions to undertake EMCR behavior.*

Drawing on basic assumptions regarding the applicability of the TPB model to predict consumers’ intentions, we expect that the abovementioned three constructs serve as core predictors of consumers’ EMCR intentions and as such shape the baseline model of this study. However, despite the theoretical and empirical robustness of the TPB, certain limitations in its predictive power of pro-environmental intentions have been recognized. For instance, it has been marked that the three key predictors allow researchers to explain only between 27% to 39% of the variance in person’s intentions (see meta-analysis by Armitage and Conner 2001; Bamberg and Möser 2007). Having acknowledged this shortcoming, scholars recommend expanding the TPB to incorporate additional components into the framework (Yuriev et al. 2020; Heidbreder et al. 2020; Ansu-Mensah and Bein 2019). Yuriev et al. (2020), in their scoping review of the TPB’s applications to the pro-environmental behavior phenomenon, convincingly argue that additional theoretically sound variables hold the potential to increase the predictive power of the TPB model. Along this line, Haugtvedt et al. (2018) suggest investigating individual differences to extend our knowledge of existing theoretical models and employ them to deepen our insight into what drives changes in attitudes and decision-making processes. In their recent work, Hopwood et al. (2022) call for in-depth explorations of such personality traits to enhance our understanding of how individuals embrace pro-environmental behavior and to provide a deeper insight into the relationship between personality traits and individuals’ pro-environmental attitudes and behaviors (Hopwood et al. 2022). Such personality traits frequently function as moderators in theoretical models (Furnham and Heaven 1999; Hansen and Sand 2008; Haugtvedt and Petty 1992).

### 2.3. Moderation Effects of the Need for Evaluation (NE) and Self-Referencing (SR)

The history and palette of notions encompassing individual differences, personality, and self-concepts is rich and colorful (Buss and Hawley 2010). Understanding the intricacy of an individual’s personality in the purchasing context has long stood high on the agenda of consumer behavior research (Baumgartner 2002; Kassarjian 1971). Building upon this scholarly tradition, this study examines the hypothesized moderating effects of two personality traits (i.e., the NE and SR) on consumers’ intention to engage in EMCR. Earlier studies in the EMCR research vein explored the moderating role of the need for status (Armstrong Soule and Sekhon 2022), need for cognition (Hüttel et al. 2020), and the self-monitoring personality traits (Ghorban Nejad and Hansen 2021). The NE and SR personality traits, distinct from the broader Big Five personality groups (i.e., extraversion, agreeableness, openness, conscientiousness, and neuroticism), offer insights into how consumers’ propensity to evaluative responding (i.e., the need for evaluation) and tendency to relate information to themselves (i.e., the self-referencing) affect the core TPB relationships articulated in the previous section. The proposed hypotheses are explicated in the paragraphs below.

#### 2.3.1. Need for Evaluation (NE)

Jarvis and Petty (1996) refer to the need for evaluation (NE) as a personality trait that reflects the extent to which individuals chronically form judgments and evaluative responses. In other words, an NE reflects the degree to which one is inclined or motivated to form evaluations about almost everything one comes across, regardless of its relevance or importance to oneself (Jarvis and Petty 1996). Specifically, individuals with observed high NE levels show more extreme evaluative responses towards a wider array of issues than their low-NE counterparts (Jarvis and Petty 1996), which plays a paramount role in individuals’ cognitive processing and decision-making (Cronley et al. 2010; Fennis and Bakker 2001).

Research across different contexts, including consumer behavior, highlights the NE’s role in attitude formation and attitude–behavior consistency (Cronley et al. 2010; Federico 2004; Bizer et al. 2004). Studies show that individuals high in the NE are more prone to engage in evaluative thinking, leading to more stable and consistent attitudes (Cronley et al. 2010; Federico 2004; Thota and Biswas 2009). Similarly, research by Federico (2004) has explored how the NE predicts and moderates the strength and extremity of attitudes. This emphasizes the role of the NE in shaping consumer judgments and engagement with marketing strategies (Thota and Biswas 2009). For instance, Cronley et al. (2010) argue that high-NE individuals are prone to form attitudes based on the processing of accessible information, which then more strongly predicts future behaviors. They found that individuals scoring higher in NE form online attitudes (i.e., on-the-spot attitudes) more than their low-NE counterparts, who form more memory-based judgments.

Thus, based on Cronley et al.’s (2010) and others’ findings, it can be argued that for individuals high in the NE, the formation of attitudes toward pro-environmental behavior is likely to result in a stronger attitude–intention relationship, especially in areas like environmental behaviors where the evaluation of the information is important to engage in EMCR behavior. This is because high-NE individuals engage more deeply with information, leading to more well-evaluated, on-the-spot attitudes that are more fundamental to their decision-making processes and add to their already existing attitudes toward pro-environmental behavior, and therefore will have an amplifying effect. However, for individuals low in the NE, their attitudes formed are more memory-based, more likely leading to a weakening attitude–intention link as they lack spontaneous evaluative responses when making judgments. More formally, this hypothesis is as follows:

**Hypothesis 4.**  *The NE strengthens the relationship between consumers’ attitudes toward pro-environmental behavior and their intention to undertake EMCR behavior. That is, for consumers who score high in the NE trait, the positive relationship between their attitudes and EMCR is amplified. This is the opposite for their low-NE counterparts.*

Furthermore, research into the NE suggests that individuals with high levels of the NE trait show a strong propensity to engage in spontaneous evaluative thinking, forming judgments based on their evaluations rather than relying heavily on external cues or influences (Bizer et al. 2004; Britt et al. 2011; Hansen and Sand 2008; Tormala and Petty 2001). This implies that high-NE individuals prioritize their internal evaluation processes over the perceived expectations of others, even in areas with social and moral implications, such as environmental sustainability. Lee’s (2021) work on the influence of the NE on attitude extremity towards hard issues such as climate change highlights that the NE influences not only the depth of attitude formation but also the reliance on internal evaluations versus external cues. Furthermore, the research by Thota and Biswas (2009) examining consumer reactions to marketing strategies emphasizes that high-NE individuals may experience irritation or dissatisfaction with messages that do not align with their evaluative criteria, including those related to environmental issues. This suggests that even consumption reduction campaigns framed within the context of societal norms could be less effective for individuals with a high NE if they perceive these messages as not aligning with their personal evaluations.

Drawing from earlier studies, it could be asserted that the relationship between consumers’ subjective norm and their intention to undertake EMCR behavior differs depending on their NE levels. High-NE consumers are more likely to have spontaneous evaluative responses and, therefore, are less likely to be influenced by their perceptions of their social pressures. When individuals are highly evaluative and base their judgments on their own evaluations and not on their perceptions of what others would think or expect, the effect of subjective norms on their intention to undertake EMCR will be weaker. By contrast, low-NE individuals are likely to avoid expressing spontaneous evaluative or polarized opinions. Accordingly, we hypothesize the following:

**Hypothesis 5.**  *The NE weakens the positive relationship between consumers’ subjective norm and their intention to undertake EMCR behavior. Specifically, for consumers who score high in the NE trait, the positive relationship between their subjective norm and intention to undertake EMCR decreases. This is the opposite for their low-NE counterparts.*

#### 2.3.2. Self-Referencing (SR)

Self-referencing (SR) refers to an individual difference by which individuals refer to their personal experiences and aspects of self when making judgments or decisions (Haugtvedt 1994). Individuals’ self-referencing relies heavily on the information stored within a person’s reference points in memory. The degree to which individuals have a propensity to self-reference varies, as individuals who score high in the SR trait are more prone to relate information to themselves than their low-SR counterparts (Haugtvedt 1994).

Research has consistently shown that SR enhances the persuasive power of messages by making them more personally relevant to the individual (Burnkrant and Unnava 1995; Escalas 2007; Haugtvedt 1994). This effect extends to advertising and marketing, where SR has been shown to increase the effectiveness of advertisements by relating the message more closely to the individual’s own experiences (Cooke et al. 2022; Lee et al. 2020; Yim et al. 2021). Research suggests that individuals with high levels of SR have more favorable attitudes toward ads or brands and higher purchase intentions than their low-SR counterparts (Hesapci et al. 2016; Martin et al. 2004; Richetin et al. 2016).

Drawing on the theoretical background and empirical findings mentioned above, it is reasonable to argue that SR could enhance the effectiveness of pro-environmental messages in motivating EMCR behavior, especially among individuals with already positive attitudes toward pro-environmental behavior. High-SR consumers make environmental concerns more personally significant, which may enhance the likelihood that a positive attitude toward the behavior translates into EMCR intentions and, therefore, amplifies this link. As such, it could be reasoned that for high-SR individuals, attitudes towards pro-environmental behavior become more important and influential in shaping intentions to reduce consumption due to the enhanced personal relevance in their judgments. Accordingly, we hypothesize the following:

**Hypothesis 6.**  *SR amplifies the relationship between consumers’ attitudes toward pro-environmental behavior and their intention to undertake EMCR behavior. Specifically, for consumers who score high in the SR trait, the positive relationship between their attitudes and EMCR is amplified. This is the opposite for their low-SR counterparts.*

Moreover, Martin et al. (2007) found that when individuals have a high internal locus of control regarding their weight (i.e., have high control over their weight) and scored high in SR, they showed a more favorable response to advertisements featuring slim models. Applying Martin et al.’s (2007) findings from the context of body image and advertising to the broader context of pro-environmental behavior such as EMCR implies that the relationship between the positive influence of how individuals see themselves as capable of undertaking pro-environmental practices (i.e., perceived behavioral control) and their EMCR intentions will be stronger for high-SR individuals. For consumers with a high SR who strongly believe in their ability to control their pro-environmental actions, their intentions to undertake consumption reduction for environmental reasons will be amplified and enhanced compared to individuals who score low in SR. In other words, when consumers assume “I know how to do it” (i.e., high PBC) since “I have done it before” (i.e., a high SR with positive experiences with consumption reduction), then the odds are higher that they intend to undertake EMCR behavior. Accordingly, we hypothesize the following:

**Hypothesis 7.**  *SR amplifies the positive relationship between consumers’ perceived behavioral control and their intention to undertake environmentally motivated consumption reduction behavior. Specifically, for consumers who score high in the SR trait, the positive relationship between their perceived behavioral control and intention to undertake environmentally motivated consumption reduction is amplified. This is the opposite for their low-SR counterparts.*

**Figure 1:**
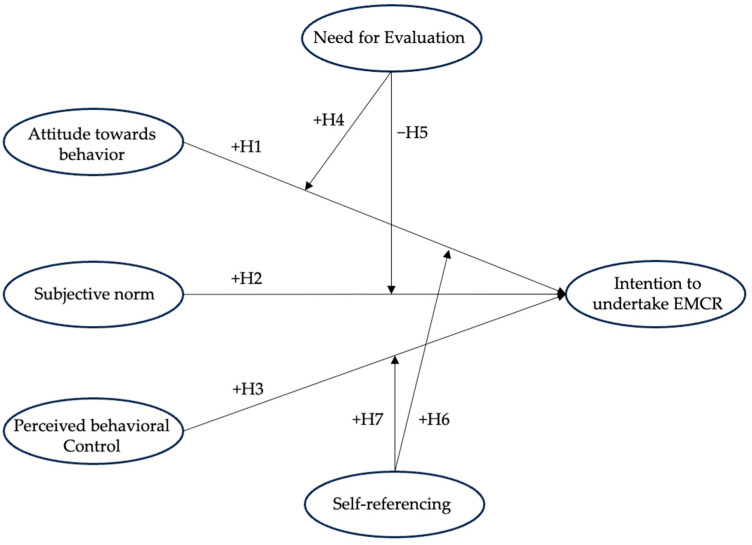
Interrelations between the TPB variables, the NE and SR moderators, and consumers’ intention to undertake EMCR with their corresponding hypotheses.

All hypothesized relationships are illustrated in Figure 1.

## 3. Materials and Method

### 3.1. Survey Sample and Data Collection Procedures

To test the hypotheses, and to recruit a nationwide sample of 226 respondents to participate in our web-based cross-sectional survey, the data collection was undertaken via an online consumer panel. We set no screening criteria in the recruitment process other than that the respondents had to be adults between the age of 18 and 64. The gender distribution among our sample was equal. The mean age of the respondents was 50 years old (M = 50, SD = 17.43). During the data collection procedure, the respondents were well-informed about the purpose of the study. Furthermore, the consumer panel ensured the anonymity and confidentiality of the data collection to respondents. The involved respondents receiving incentives such as points in return for their participation, which they could redeem for gift cards or vouchers.

Our research was carried out in Norway, a country with a relatively small population but whose residents’ high levels of consumption significantly add to global carbon emissions (Global Footprint Network 2022). Investigating the determinants of consumers’ EMCR intentions is important in affluent societies, where consumption levels exceed environmental limits (Global Footprint Network 2022; Spaiser et al. 2019; Steffen et al. 2018). Notably, some high-consumption developed countries may not rank as major polluters since the emissions associated with their consumed goods are often produced in less developed nations (Global Footprint Network 2022; Spaiser et al. 2019; Steffen et al. 2018).

### 3.2. Questionnaire Design and Measurements

In developing the baseline constructs for the TPB, we adapted the measurement items from scales previously validated by Ajzen (2002). Similarly, we adapted the Need for Evaluation Scale (NES) from the 16-item scale developed and validated by Jarvis and Petty (1996) and the 8-item Propensity to Self-referencing (SR) scale developed and validated by Haugtvedt et al. (2004). Both selected moderators’ scales are internally consistent with high-reliability scores and possess satisfactory convergent and discriminant validity properties (Haugtvedt et al. 2004; Jarvis and Petty 1996). Furthermore, we employed Norwegian versions of these scales which have been validated in prior research in the Norwegian language (Hansen et al. 2011; Hansen and Sand 2008; Vabø and Hansen 2016).

In contrast to the methodologies employed in previous studies (e.g., Ghorban Nejad and Hansen 2021; Joanes 2019), our approach to measuring the dependent variable differed as we have not paid attention to a specific category of products or services associated with consumption reduction. Instead, we examined an aggregated set of EMCR intentions, including reduction through a range of practices like transport, food, and plastic use, all known for their significant environmental impacts (Sandberg 2021). This broader approach aligns with the findings of previous research, which has established the generalizability of EMCR as a composite construct (Egea and de Frutos 2013; Lasarov et al. 2019; Richetin et al. 2012). This construct captures a variety of consumption reduction behaviors across different domains rather than isolating a single category. To measure the intention to engage in EMCR behaviors, we adapted measurement items from Derdowski et al. (2020), applying slight modifications to the wording to better suit the context of our study. All items were measured using a 7-point Likert scale, ranging from 1 (“completely disagree”) to 7 (“completely agree”). Detailed descriptions of the adapted measurement items are provided in Appendix A.

### 3.3. Analytical Strategy

All proposed hypotheses were analyzed using the Statistical Package for Social Sciences (SPSS) and AMOS software, both at version 29. A covariance-based structural equation modeling (SEM) approach was employed to analyze the survey data. Preliminary analyses were done in SPSS to check for the normality of the data (see Table 1). This was checked using the skewness and kurtosis values of all the latent variables. According to Tabachnick et al. (2013), skewness and kurtosis values of variables between −2 and +2 largely indicate a normal distribution. Regarding missing values, the design of the survey required respondents to answer each question before moving on to the next one, effectively eliminating any missing values.

Confirmatory factor analysis (CFA) was then conducted in AMOS to assess the factor structure of all the variables. The CFA was used to check the factor loadings of items and the reliability as well as the validity of the variables with regard to the data. Based on the CFA results, structural models were then developed. The initial structural model comprised the predictors and the outcome variable. The moderating variables were added next, and then the interaction terms were included. These interaction terms were developed by first standardizing (Z-scores) the items (observed variables) of the predictors and moderators, and subsequently computing their combinations at the latent level. This was done in SPSS. Model fitness was ascertained for both CFA and structural models based on the recommendations of Hu and Bentler (1999). To better illustrate the moderation effects, an online statistics tool package in Microsoft Excel developed by Gaskin (2016) was used to generate the graphs. For each graph, the unstandardized beta values from the final structural model for each specific predictor, moderator, and outcome were input into the package on interaction effects, thus automatically generating the graphs.

## 4. Results

### 4.1. Confirmatory Factor Analysis (CFA)

As the variables included in the model had previously been validated in other studies, we initiated the analysis with confirmatory factor analysis (CFA) to verify the factorial structure of model’s variables. The CFA allowed for testing the psychometric properties of latent constructs and resulted in a two-step model adjustment for the optimal model fit. At first, all items with low factor loadings (below 0.5) were excluded, yet at least two items per construct were retained, as per Kenny’s (2016) guidelines. This resulted in a fine-tuned model where nine items from the NE scale and one item from the attitude scale were removed, and no alterations were applied to the other constructs. Next, items within the same construct that were marked with high modification indices were addressed by covarying their error terms (See Appendix B). According to Hu and Bentler’s (1999) recommendations, the obtained model demonstrated a satisfactory fit: CMIN/DF = 1.76, CFI = 0.91, TLI = 0.90, RMSEA = 0.058), with factor loadings between 0.58 and 0.87.

### 4.2. Validity and Reliability

For convergent validity, the average variance extracted (AVE) values were examined. Except for PBC and EMCR, all constructs in this study showed AVE values greater than 0.50, with PBC and EMCR approaching this benchmark (see, Table 1). Of relevance, Malhotra and Dash (2011) have cautioned that the threshold value of 0.50 might be overly strict for certain constructs, especially for those like EMCR that are emerging and require a systematic psychometric standardization.

Discriminant validity was evaluated using the Fornell–Larcker criterion, which is supported when the square root of a construct’s AVE is greater than identified correlations between that construct and others (Fornell and Larcker 1981). In terms of discriminant validity, all constructs were distinct from each other, with the exception of the relationship between EMCR and attitudes toward behavior (AT), where the close relationship could be attributed to the phrasing of the items (see Appendix A for the wording of these measurement items). For all other variables, the square roots of the AVEs exceeded estimated correlations among the constructs, thereby confirming discriminant validity as per Fornell and Larcker’s (1981) criterion. Reliability was assessed through composite reliability values (CRVs), with a value of 0.70 or above indicating an acceptable internal consistency of a scale. The results of the validity and reliability assessments are summarized in Table 2.

### 4.3. Structural Model

Following the CFA, a structural model was estimated that incorporated all latent variables and their associated items retained from the CFA. Additionally, the effect of age (in years) on EMCR was controlled for, as previous studies revealed a significant relationship between respondents’ age and their intention to reduce consumption (Culliford and Bradbury 2020). The final model achieved very good fit indices (CMIN/DF = 2.615; GFI = 0.982; CFI = 0.974; RMSEA = 0.085), surpassing the benchmarks established by Hu and Bentler (1999).

#### 4.3.1. Main Effects

**Figure 2:**
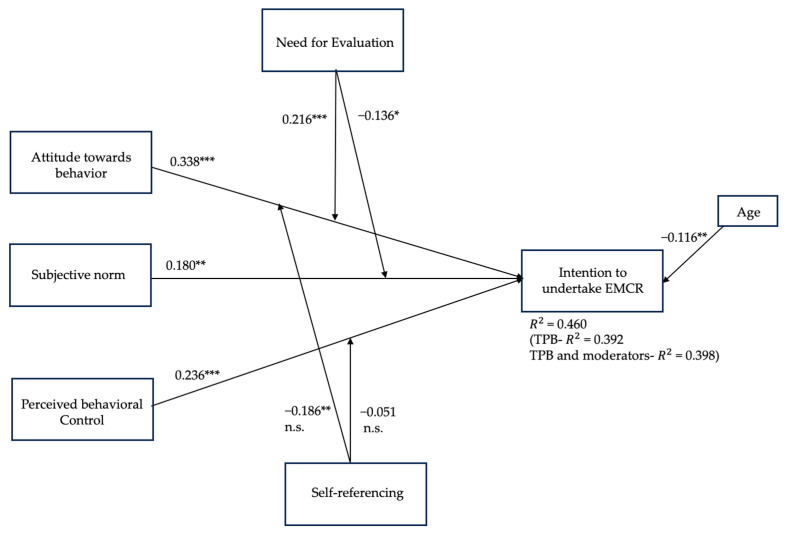
The final structural equation model (SEM) with standardized beta values. Notes: *** *p* ≤ 0.001; ** *p* ≤ 0.01 and * *p* ≤ 0.05; Results in parentheses represent $R^{2}$ value with the core TPB predictors only and with the TPB and moderators’ independent effects; n.s. = not supported.

The results depicted in Figure 2 indicate that all three TPB variables, namely, attitude toward behavior (β = 0.338, *p* ≤ 0.001), subjective norm (β = 0.180, *p* ≤ 0.01), and perceived behavioral control (β = 0.236, *p* ≤ 0.001), have positive and significant associations with consumers’ intention to undertake EMCR behavior (R2 = 0.39, for the core TPB predictors). This implies that when consumers have a positive attitude toward a pro-environmental behavior, experience social pressures from significant others, and believe to have control over the pro-environmental behavior, then they are more likely to express intention to undertake EMCR to benefit the environment. Thus, Hypotheses 1–3 regarding the main effects were all confirmed.

#### 4.3.2. Moderating Effects

**Figure 3:**
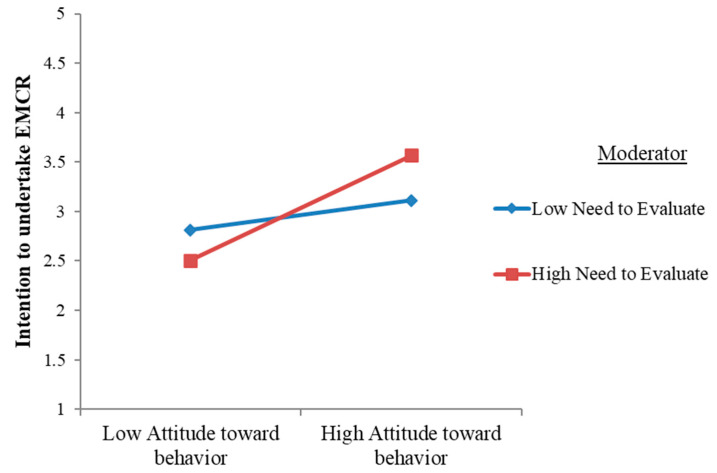
Moderation plot for attitude and need for evaluation.

The inclusion of moderating variables into the model revealed that consumers’ NE (β = 0.037, *p* = 0.481) and SR (β = −0.009, *p* = 0.875) as independent predictors had no significant direct effects on the intention to engage in EMCR behavior. For the moderation analysis, “low” and “high” levels of the variables are defined as one standard deviation (SD) below and above the mean of the moderator. Specifically, individuals with low levels are less likely to engage in evaluative thinking (for the NE) or less likely to relate information to themselves (for SR), while those with high levels are more likely to do so. The results show that the estimate encapsulating the moderating effect of the NE (i.e., the Hypothesis 5) was statistically significant (β = 0.216, *p* ≤ 0.001). This implies that for consumers who are highly evaluative and constantly shape evaluative responses to their surroundings, the positive relationship between their attitudes toward pro-environmental behavior and their intention to undertake EMCR was strengthened as compared to those with a low NE score. Thus, H4 was corroborated (please refer to Figure 3 for illustration).

**Figure 4:**
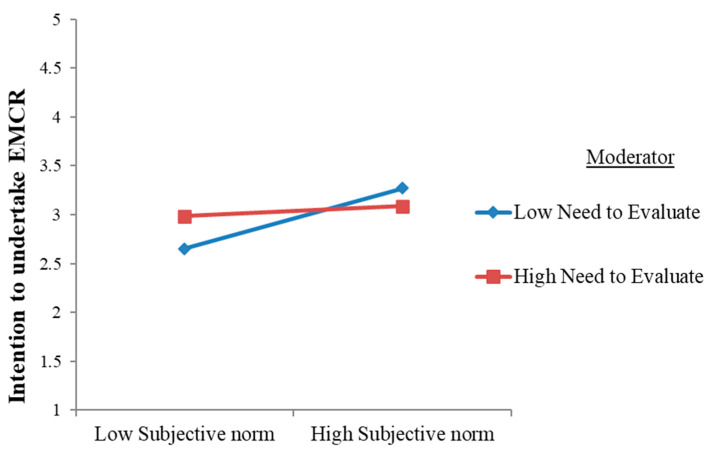
Moderation plot for subjective norm and need for evaluation.

Furthermore, the estimated coefficient that illustrates the NE’s moderation of the relationship between subjective norm and EMCR intentions was negative and significant (β = −0.136, *p* ≤ 0.05). This further implies that when consumers scored high in the NE personality trait, the positive relationship between their subjective norm and their intention to undertake EMCR got weaker as compared to those with a low NE score. Thus, H5 was also corroborated (please refer to Figure 4 for illustration).

The analyses further demonstrated that the SR personality trait holds the potential to weaken the relationship between the attitude toward the pro-environmental behavior and EMCR intentions (β = −0.186 and *p* ≤ 0.01). While contrary to the presented rationale for Hypothesis 6, this finding suggests that when consumers score high in SR, the positive relationship between their attitude toward the behavior and their intention to undertake EMCR gets weaker as compared to those with a low SR score. Thus, despite its statistical significance, H6 was not supported.

Lastly, the conducted tests revealed that SR does not moderate the relationship between respondents’ perceived behavioral control and EMCR intentions (β = −0.051 and *p* > 0.05). Thus, H7 was not supported due to a lack of statistical significance. The summary of the results with standardized parameter estimates, statistical significance level, and for all proposed hypotheses is presented in Table 3.

Finally, to assess whether the extended model that incorporates moderating variables offers a more comprehensive explanation of the variance in the intentions to engage in EMCR compared to the core TPB, or the core TPB and the moderators, a simple auxiliary test was conducted. Out of the three scrutinized SEM models, the first one solely included the TPB variables (i.e., ATT, SN, PBC, and intention to EMCR), the second one the additive effects of the TPB variables and the two moderators, while the third one was extended by inspecting NE and SR with their accompanying moderating effects. Accordingly, the estimated R-square values were equal to 0.392, 0398, and 0.460. This implies that the inclusion of the NE and SR personality differences as moderators enhances the model’s explanatory power by seven percentage points, raising from 39% to 46%, respectively.

## 5. Discussion

According to Gessert and Klein (2015), we live in a failed economic system that is persistently and inevitably pushing humanity towards the most profound environmental threat we have ever faced. With the escalating environmental problems and increasing material footprints all over the world and in affluent societies in particular (UN DESA 2023), forward-thinking ideas that will take us off the beaten path of, for instance, sustainable consumption research, grow in demand (Diesendorf and Taylor 2023; McPhearson et al. 2021). This paper aims to put the spotlight on the important topic of consumption reduction by understanding individuals’ intentions to engage with consumption reduction practices seen through the lens of an extended TPB model. Consistent with prior research (Ansu-Mensah and Bein 2019; Mattia et al. 2019), our findings illustrate the theory of planned behavior’s applicability in predicting respondents’ EMCR intentions. In line with the emerging sustainable transformations degrowth research that advocates for significant reductions in energy and resource usage (Islar et al. 2024; Schmelzer et al. 2022), the TPB theory seems valuable for scholars, policymakers, and marketers committed to promoting EMCR.

Furthermore, to realize the full potential of TPB for explaining and/or predicting consumers’ EMCR intentions, it is advisable for the aforementioned interest groups to consider how individual differences might affect the feasibility of consumption reduction initiatives. The findings from this study imply that for individuals who inherently engage in high levels of evaluative thinking (i.e., those with a high need for evaluation) the positive relationship between their attitudes toward pro-environmental behavior and their intention to undertake EMCR was strengthened as compared to those with a low NE score. This moderation effect suggests, thus, that an individual’s depth and quality of evaluative processing play an important role in translating positive pro-environmental attitudes into concrete intentions to reduce consumption. On top of that, this study reveals an interesting interaction between personal evaluative processes and perceived social influences. Essentially, obtained results indicate that when consumers scored highly in the NE personality trait, the positive relationship between their subjective norm and their intention to undertake EMCR was impaired as compared to those with a low NE score. This observed effect challenges the traditional assumptions about the perceived social pressures on pro-environmental behavior (Ajzen 1991) and further suggests that individuals with a high NE prioritize their own judgments over the perceived social pressures of significant others when considering EMCR behavior. This rationale aligns with Lee’s (2021) findings, which demonstrated that high-NE individuals rely heavily on internal evaluations when encountering debatable topics such as the one of climate change.

Contrary to the logic articulated to support Hypothesis 5, the yielded evidence shows that a high self-referencing individual trait impairs the positive relation between one’s pro-environmental attitude and an EMCR intention. This unexpected discovery could be reasoned, for example, through the work of Lasarov et al. (2019), who contended that consumers remain skeptical about EMCR because it requires high levels of commitment and sacrifice to make significant changes in one’s behavior. As such, it could be argued that individuals with a strong tendency to relate information to their personal experiences (high SR) may prioritize personal relevance and immediate benefits (in line with the self-interest motive (Miller 1999) over environmentally motivated consumption patterns that demand significant efforts and/or sacrifice (like in the case of EMCR). In a related vein, prospects of consumption reduction could be associated as well with the loss aversion bias (Kahneman et al. 1991), which essentially states that the pain of losing something is more acute than the pleasure of gaining something of equal value. While the effect of loss aversion on pro-environmental decisions has extensively been studied before (Homar and Cvelbar 2021), the results of this study imply that its gravity could be even more pronounced among high-SR consumers. Lastly, the hypothesized SR’s moderating impact on the positive relationship between one’s perceived behavioral control on EMCR intentions was not supported. To rationalize such a result, one needs to recognize that the aforesaid proposition rests on the assumption that individuals possess some kind of experience or information on EMCR available in their memory that will allow them to self-reference and consider aspects of their behavioral control. However, these psychological effects are unlikely to materialize if, due to, e.g., loss aversion or a self-interest motive, one lacks a consumption reduction point of reference. Additionally, as consumers are prone to the availability bias (i.e., they tend to rely on the events readily available in their minds (Korteling et al. 2023), these past experiences should preferably be rather recent and frequent (Hansen et al. 2011), yet more often than not, this is not the case for EMCR (Lasarov et al. 2019). Thus, taken together, the described findings mark the potential and importance of further investigations into how different levels of self-reference affect and relate to individuals’ environmentally motivated consumption reduction intentions and behaviors. Considering the theoretical contribution of the current article, our proposed model could be further expanded to also comprise individuals’ cognitive ability, such as fluid intelligence, which is the ability to think abstractly, reason, and solve problems (Kyllonen and Kell 2017), or factual knowledge, to see how these cognitive abilities interact with personality dimensions like the NE and SR. This would deepen the theoretical understanding of pro-environmental behaviors in general and consumption reduction in particular. This further provides more tailored insights for interventions aimed at promoting sustainable consumption, particularly among individuals with varying cognitive capabilities.

From a practical perspective, consumers’ profiles have long been studied with a range of demographic, psychological, socioeconomic, geographic, and cultural measures to predict their decisions and behaviors. For instance, Schwepker and Cornwell (1991) summarized personality variables investigated in 17 studies conducted in in the 1970s and ‘80s on environmentally concerned consumers. Today, Artificial Intelligence (AI) technology allows us to tailor customized and personalized offerings based on available consumer data to leverage marketing practices for social and environmental good (Hermann 2022). Along this line, insights deriving from this study could be of interest to the broader group of consumers, social marketers, and policymakers in the consumption reduction field, considering its implications, such as tailoring AI-powered environmental messages and campaigns that would take into account individual personality differences to enhance the effectiveness of consumption reduction strategies. Online platforms, for example, could employ machine learning algorithms to profile users based on their interactions (Simester et al. 2020) and so generate personalized content that would cater to the evaluative tendencies of high-NE individuals or the self-related preferences of those high in SR. This approach challenges the efficacy of generic “save the planet” environmental campaigns and highlights the considerable potential of future consumption reduction policies and initiatives that account for individual differences aimed at encouraging and promoting consumption reduction practices to benefit the environment.

### Limitations and Future Avenues

This study scrutinized consumers’ intentions to undertake EMCR through the lens of the extended TPB, without recording actual EMCR behaviors. Admittedly, the phenomenon of intention–behavior gap has been widely documented in academic literature (Sheeran and Webb 2016), including in studies into green consumption (Nguyen et al. 2019). On top of that, whereas the TPB postulates that one’s attitudes, subjective norms, and perceived behavioral control are significantly associated with one’s behavior through an indirect effect of formed intentions (Ajzen 1991), the cross-sectional research design adopted for this project prevents us from drawing causal inferences. Thus, to address the abovementioned shortcomings, future studies are encouraged to employ longitudinal and experimental designs to further explore emerging causal relationships and overcome the problem of social desirability bias that oftentimes is observed in studies based on self-reported measures (Nederhof 1985). Moreover, conducting research in Norway (a country with relatively high standard of living and high consumption levels) may affect the generalizability of the findings. Future studies are encouraged to expand this research beyond developed countries. Further, other socio-demographic factors, such urban or rural environments, may play important roles in consumers’ consumption levels that could be explored in future studies. We also acknowledge the complexity of SEM models with a moderate sample size. However, as indicated by several scholars (e.g., MacCallum et al. 1999; Wolf et al. 2013), one size does not fit all, and more is not always better. Following Kline (2023), our study, to a large extent, showed a normal distribution and therefore the sample size was adequate; however, we suggest future research to employ larger sample sizes. Regarding the moderating effects, we acknowledge that one way to gain further insights on the moderating role of individual differences would be through the application of more advanced statistical modeling approaches such as Johnson–Neyman–Intervall (JNI). Future research may employ such approaches to allow more detailed reports on in which ranges of the moderator the moderation effects occur.

Additionally, our focus on two personality differences (i.e., the need for evaluation and self-referencing) was driven by their theoretical relevance to the submitted research objectives and unique contribution to previous environmental consumption studies. However, acknowledging the complexity of consumer pro-environmental behaviors and richness of the self-related concepts (Sirgy 1982; Thagard and Wood 2015 refer to eighty phenomena), future endeavors could extend the existing scholarly horizon by considering a broader range of personality traits or cognitive abilities to fully understand their impact and relation to EMCR. Incorporating such cognitive factors may further illuminate how individuals interpret and act upon environmental messages, potentially enhancing the predictive power of the TPB framework in this context.

## 6. Conclusions

Consumption reduction offers a somehow disputable yet perfectly viable path to ensure global environmental sustainability. Emerging strands of research and practice support the notion that shifting to green consumption is not the only way to meet United Nations’ Sustainable Development Goals (SDGs), including SDG 12, which revolves around responsible consumption and production. This study provides evidence supporting the applicability of the extended theory of planned behavior to model and understand consumers’ intention to undertake consumption reduction behaviors. Identified moderating effects of individual differences, i.e., the need for evaluation and self-referencing traits, bring insights that offer value to policymakers and social marketers who may strive to encourage individuals to consume less for the benefit of the natural environment.

## Figures and Tables

**Table 1:** Psychometric properties of the measures for all the study variables.

Variable	M	SD	Skewness	Kurtosis
Attitude toward behavior (AT)	4.86	0.89	−0.039	0.012
Subjective norm (SN)	4.57	1.29	−0.241	−0.003
Perceived behavioral control (PBC)	4.37	1.26	−0.068	−0.227
Self-referencing (SR)	5.17	1.03	−0.608	1.088
Need for evaluation (NE)	3.94	0.70	0.087	0.058
EMCR	4.02	1.48	0.132	−0.686

**Table 2:** Assessment of reliability and validity of retained items.

Variable	# of Items	CRV	AVE	SR	NE	AT	SN	PBC	EMCR
SR	8	0.894	0.517	**0.719**					
NE	7	0.892	0.545	0.243	**0.738**				
AT	3	0.848	0.652	0.197	0.009	**0.808**			
SN	3	0.836	0.631	0.234	0.204	0.654	**0.794**		
PBC	4	0.763	0.448	0.105	0.141	0.584	0.590	**0.669**	
EMCR	5	0.789	0.430	0.035	0.094	0.680	0.581	0.545	**0.656**

Notes: CRV = composite reliability value; AVE = average variance extracted; Estimates on the diagonal (in bold) = square roots of the AVEs; SR = self-referencing; NE = need for evaluation; AT = attitudes toward pro-environmental behavior; SN = subjective norm; PBC = perceived behavioral control; EMCR = intention to undertake EMCR behavior.

**Table 3:** Summary of the results. Structural model.

Hypotheses	Std. β	*p* Value	Results
H1. Attitude towards pro-environmental behavior→ EMCR intentions	0.338	0.000	Supported
H2. Subjective norm→ EMCR intentions	0.180	0.006	Supported
H3. Perceived behavioral control →EMCR intentions	0.236	0.000	Supported
H4. Attitude towards behavior × need for evaluation → EMCR intentions	0.216	0.000	Supported
H5. Subjective norms × need for evaluation → EMCR intentions	−0.136	0.025	Supported
H6. Attitude towards behavior × self-referencing → EMCR intentions	−0.186	0.003	Not supported
H7. Perceived behavioral control × self-referencing → EMCR intentions	−0.051	0.407	Not supported

**Figure A1:**
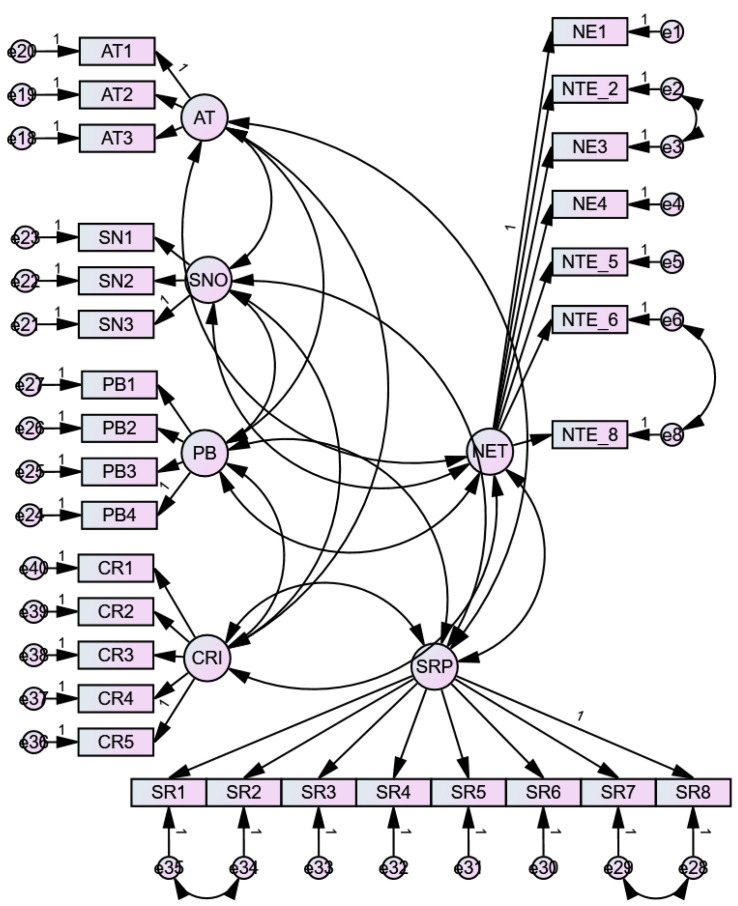
CFA path diagrams.

## Data Availability

Data will be made available on request.

## Appendix A

**Table A1:** Measurement items (translated into English).

Measurement Items
Attitude toward pro-environmental behavior (ATT) ATT1. That we all choose to act more sustainably will be very good for the environment.
ATT2. I have a positive impression of the environmentally friendly alternatives I encounter on a daily basis.
ATT3. The fact that something you are considering buying or doing is environmentally friendly adds an extra important quality dimension.
ATT4. I think the current environmental regulations are more than strict enough.
Subjective norm concerning pro-environmental behavior (SN) SN1. Among those I associate with, there is a widespread belief that one should choose environmentally friendly alternatives.
SN2. People who are important to me are concerned with acting for the good of the environment.
SN3. My friends and family think we should act in an environmentally friendly way when we have the opportunity.
Perceived behavioral control concerning pro-environmental behavior (PBC) PBC1. There are so many opportunities to act environmentally friendly that there is no problem.
PBC2. It is not difficult to make environmentally friendly choices on a daily basis.
PBC3. It does not cost anything extra to act environmentally friendly.
PBC4. I have no problem paying what it costs to act environmentally friendly.
Self-referencing (SR) SR1. Understanding new things and unfamiliar situations always helps to think about my past experiences.
SR2. It is easier to learn new things if they can be related to ourselves and our experiences.
SR3. When I read stories, I am often reminded of my own experiences from similar situations.
SR4. I often use past experiences to remember new information.
SR5. I think it is easier to evaluate anything if it can be related to ourselves and our experiences.
SR6. I always think about how my surroundings affect me.
SR7. In conversations with others, I constantly discover that I think of my own experiences when they describe theirs.
SR8. If I have to describe thoughts and ideas to others, it often happens that I use my own experiences as examples.
Need-to-evaluate (NTE) NTE1. I form opinions about everything.
NTE2. I prefer to avoid taking extreme opinions. (reverse coded)
NTE3. It is very important to me to hold strong opinions.
NTE4. I want to know exactly what is good and bad about everything.
NTE5. I often prefer to remain neutral about complex issues. (reverse coded)
NTE6. If something does not affect me, I do not usually determine if it is good or bad. (reverse coded)
NTE7. I enjoy strongly liking and disliking new things.
NTE8. There are many things for which I do not have a preference. (reverse coded)
NTE9. It bothers me to remain neutral.
NTE10. I like to have strong opinions even when I am not personally involved.
NTE11. I have many more opinions than the average person.
NTE12. I would rather have a strong opinion than no opinion at all.
NTE13. I pay a lot of attention to whether things are good or bad.
NTE14. I only form strong opinions when I have to. (reverse coded)
NTE 15. I like to decide that new things are really good or really bad.
NTE16. I am pretty much indifferent to many important issues. (reverse coded)
Intention to undertake Environmentally Motivated Consumption Reduction (CR) CR1. In the future, I will try to choose bicycle or public transport instead of car or taxi.
CR2. When I travel in the future, I will choose train or bus instead of flying.
CR3. Staying at home rather than traveling is something I will choose to reduce the number of flights.
CR4. Choosing items with a packaging that produces less plastic waste is something I will prioritize in the future.
CR5. I will eat less meat and more plant-based food in the future.

## Appendix B

**Table A2:** Range of factor loadings and McDonald’s Omega.

Variable	Range of Factor Loadings	Omega Ω
Attitude (AT)	0.71–0.87	0.853
Subjective norm (SNO)	0.74–0.83	0.831
Perceived Behavioral Control (PB)	0.62–0.74	0.745
Need-to-evaluate (NET)	0.62–0.84	0.904
Self-referencing (SRP)	0.59–0.85	0.898
Intention to undertake EMCR (CRI)	0.58–0.74	0.788

Notes: SRP = self-referencing; NTE = need for evaluation; AT = attitudes toward pro-environmental behavior; SNO = subjective norm; PB = perceived behavioral control; CRI = intention to undertake EMCR behavior.

**Table A3:** Bivariate correlation analysis after CFA.

Variable	AT	SNO	PB	NTE	SRP	CRI
Attitude (AT)	1					
Subjective norm (SNO)	0.56 **	1				
Perceived behavioral control (PB)	0.48 **	0.49 **	1			
Need to evaluate (NTE)	0.01	0.16 *	0.11	1		
Self-referencing (SRP)	0.18 **	0.19 **	0.07	0.27 **	1	
Intention to undertake EMCR (CRI)	0.54 **	0.49 **	0.46 **	0.08	0.03	1

Notes: ** correlation is significant at 0.01 level, and * correlation is significant at 0.05 level. SRP = self-referencing; NTE = need for evaluation; AT = attitudes toward pro-environmental behavior; SNO = subjective norm; PB = perceived behavioral control; CRI = intention to undertake EMCR behavior.
